# Long term impact of CTLA4 blockade immunotherapy on regulatory and effector immune responses in patients with melanoma

**DOI:** 10.1186/s12967-018-1563-y

**Published:** 2018-07-04

**Authors:** Janet Retseck, Alexis Nasr, Yan Lin, Huang Lin, Prateek Mendiratta, Lisa H. Butterfield, Ahmad A. Tarhini

**Affiliations:** 1 University of Pittsburgh Cancer Institute, Pittsburgh, USA; 2 Department of Hematology and Oncology, Cleveland Clinic Taussig Cancer Institute, 9500 Euclid Ave CA6-157, Cleveland, OH 44195 USA; 3 Departments of Medicine, Surgery and Immunology, University of Pittsburgh School of Medicine, UPMC Hillman Cancer Center, Pittsburgh, USA; 4 Case Comprehensive Cancer Center, Cleveland, USA

**Keywords:** Melanoma, CTLA4, Ipilimumab, Regulatory T cells, MDSC, Tumor antigens

## Abstract

**Background:**

We previously reported early on-treatment significant modulation in circulating regulatory T cell (Treg), myeloid derived suppressor cells (MDSC) and antigen-specific type I CD4+ and CD8+ T cells that correlated with clinical outcome in regionally advanced melanoma patients treated with neoadjuvant ipilimumab. Here, we investigated the long term immunologic impact of CTLA4 blockade.

**Methods:**

Patients were treated with ipilimumab given at 10 mg/kg IV every 3 weeks for 2 doses bracketing surgery. Blood specimens were collected at baseline and during treatment for up to 9 months. We tested immune responses at 3, 6, and 9 months utilizing multicolor flow cytometry. We compared frequencies of circulating Treg and MDSC on-study to baseline levels, as well as frequencies of CD4+ and CD8+ T cells specific to shared tumor-associated antigens (Gp-100, MART-1, NY-ESO-1).

**Results:**

Levels of Treg significantly increased when measured at 6 weeks following ipilimumab but returned to baseline by 3 months, with no significant difference in Treg levels between relapsed and relapse-free groups at 3, 6 or 9 months. However, lower baseline levels of circulating Treg (CD4+CD25hi+CD39+) were significantly associated with better relapse free survival (RFS) (p = 0.04). Levels of circulating monocytic HLA-DR+/loCD14+ MDSC were lower at baseline in the relapse-free group and further decreased at 6 weeks, though the differences did not reach statistical significance including measurements at 3, 6 or 9 months. We detected evidence of type I (interferon-γ producing), activated (CD69+) CD4+ and CD8+ antigen-specific T cell immunity against cancer-testis (NY-ESO-1) as well as melanocytic lineage (MART-1, gp100) antigens in the absence of therapeutic vaccination. These responses were significantly boosted at 6 weeks and persisted at 3, 6 and 9 months following the initiation of ipilimumab.

**Conclusions:**

Lower Treg levels at baseline are significantly associated with RFS and increased Treg frequency after CTLA4 blockade was only transient. Lower MDSC was also associated with RFS and MDSC levels were further decreased after ipilimumab. Tumor specific effector immune responses are boosted with CTLA4 blockade and tend to be durable.

*Trial registration* ClinicalTrials.gov Identifier: NCT00972933

## Background

Clinically detectable locally and regionally advanced melanoma has a 5-year relapse rate that exceeds 70% at 5 years [1–4]. The development of local or regional recurrence after initial surgical management portends an even poorer prognosis [5–7]. In the Melanoma Intergroup Surgical Trial, a local recurrence was associated with 5 and 10 year survival rates of 9–11% and 5%, respectively [6]. To date, interferon-alfa (IFN), ipilimumab (ipi) and nivolumab have achieved regulatory approvals for the adjuvant treatment of high-risk melanoma following surgical management, while the combination of dabrafenib and trametinib was granted a breakthrough therapy designation by the Food and Drug Administration for stage III melanoma with a BRAF V600 mutation [1]. Treatment with high-dose IFN was shown to improve relapse-free survival (RFS) in three cooperative group studies [8–10], and overall survival (OS) in two out of the three versus observation [9], and versus the GMK ganglioside vaccine [8]. Adjuvant ipilimumab at 10 mg/kg was approved by the FDA in 2015 after improved RFS compared to placebo in stage III melanoma was demonstrated in EORTC 18071 trial [11]. A recent update from this trial has also reported a significant OS benefit [12].

Ipilimumab is a monoclonal antibody directed against the immune checkpoint molecule CTLA-4. It was approved by the FDA in 2011 at the dose of 3 mg/kg for use in patients with inoperable advanced melanoma [13]. CTLA-4 is expressed on activated T cells under physiologic conditions and plays a role in downregulating the immune response and protecting against autoimmunity [14, 15]. While CTLA-4 is upregulated on activated T cells, it is expressed constitutively on CD4+CD25+ Treg, which in turn suppress effector T cells [16, 17]. One means of tumor escape from immune response is through recruitment of Treg to the tumor microenvironment, which then can recognize tumor-associated antigens and expand [18]. CTLA-4 blockade with ipilimumab should lead to suppression of Treg function, allowing an immune response to tumor antigens to emerge and expand.

**Fig. 1:**
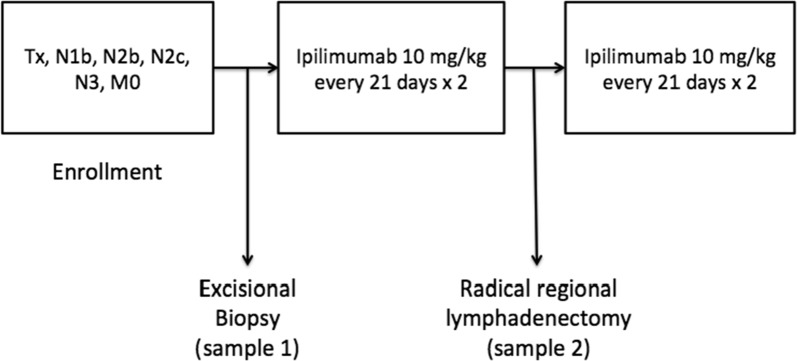
Neoadjuvant ipilimumab study design. Patients with locally and/or regionally advanced melanoma were enrolled and underwent excisional biopsy. They then received 2 cycles of ipilimumab 10 mg/kg every 21 days. A radical definitive surgery was then performed. Patients received 2 additional cycles of ipi 10 mg/kg every 21 days. Blood specimens (serum/peripheral blood monitoring) were collected at baseline, 6 weeks, 3, 6, and 9 months

We have previously reported the results of short-term (at baseline then at 6 weeks following the initiation of ipilimumab) testing of the immune response in the circulation and in the tumor microenvironment in patients receiving neoadjuvant ipilimumab for locally advanced melanoma [19]. In this study, 35 patients with resectable Stage IIIB/C melanoma received up to 2 doses of neoadjuvant ipi at 10 mg/kg IV every 3 weeks in the absence of limiting toxicity. Tumors were resected and patients received up to 2 additional doses of ipi (Fig. 1). Of 33 evaluable patients, 2 had a complete response, 1 a partial response, 21 had stable disease, and 8 had progression of disease on PET/CT 6 weeks after the initiation of ipi (by RECIST; unconfirmed). Median PFS was 10.8 months (95% CI 6.2–19.2). Peripheral blood and tumor immune response assessments were performed at 6 weeks by multicolor flow cytometry on 27 patients with available samples. We saw a significant increase in circulating Treg from baseline, with a greater increase in Foxp3+ Treg significantly associated with PFS (p = 0.034). We also observed a decrease in percentage of all populations of MDSC from baseline. The greatest decrease was seen for HLA DR+/lo CD14+ MDSC, while a greater decrease in Lin1−/HLA DR−/CD33+/CD11+ MDSC was associated with PFS (p = 0.03). In addition, there was evidence of type I (IFN gamma-producing), fully activated (CD69) CD4+ and CD8+ antigen specific T-cell immunity against cancer-testis (NY-ESO-1) and melanocytic lineage (MART-1 and gp100) antigens in the absence of therapeutic vaccine. The response was significantly potentiated at 6 weeks following ipilimumab. We hypothesized that the early on-treatment Treg increases seen were transient given the reported association with clinical benefit while the expected impact on MDSC and effector T cells was durable beyond the early-on treatment observations.

The present study reports results of long-term monitoring of Treg, MDSC, and tumor antigen-specific T cell responses at 3, 6, and 9 months following treatment relative to the previous changes seen at 6 weeks.

## Methods

### Patients

Ethics statement: the study was approved by the University of Pittsburgh Institutional Review Board (IRB; IRB# PRO09010033). All patients had a University of Pittsburgh IRB approved written informed consent obtained. The study was conducted in accordance with the principles expressed in the Declaration of Helsinki. Patient characteristics are shown in Table 1. Eligible patients were 18 years or older and had clinically detectable locally and/or regionally advanced melanoma (cutaneous, mucosal or unknown primary).

**Table 1:** Patient demographics and disease characteristics (n = 31)

Variable	Number of patients (%)
Median age, years (range)	54 (40–87)
Cutaneous primary	25 (81)
Unknown primary	1 (3)
Mucosal	5 (16)
Gender	
Female	10 (32)
Male	21 (68)
Performance status	
0	10 (32)
1	21 (68)
Prior adjuvant high-dose IFN	10 (32)
AJCC stage	
IIIB	6 (19)
IIIC	25 (81)

### Laboratory methods

Multicolor flow cytometry was used to compare cell subset phenotypes on thawed patient peripheral blood mononuclear cells (PBMC), with healthy donor controls, run according to laboratory SOPs. Treg were defined as in the previous study (19) to allow direct comparison, as CD4+CD25+FOXP3+ or CD4+CD25hi+CD39+ cells, to incorporate the candidate functional marker CD39. MDSC were defined as cells expressing Lin-neg/HLA-DR−/CD33+/CD11b+ in either a “lymphocyte” (small FSCxSSC) gate, or in a “monocyte” (larger FSCxSSC) gate, and as HLA-DR+/lo CD14+ cells in a large gate. We compared frequencies of circulating Treg and MDSC on-study to baseline levels. We also tested the frequencies of CD4+ and CD8+ T cells specific to shared tumor-associated antigens (Gp-100, MART-1, NY-ESO-1) utilizing overlapping peptide libraries (15-mer peptides overlapping by 4) and a short (4–5 h) in vitro culture to identify activated (CD69+) and cytokine producing (intracellular IFNγ+) T cells. Detailed methods were described previously [19].

### Statistics

The association of cell frequencies in relation to the probability of the event of melanoma relapse at 9 months was assessed. Patients were divided into two groups: relapsed (relapsed or died before 9 month) and non-relapsed (relapse-free by 9 months). There is no censoring during the first 9 months post-treatment. The level of each marker at baseline, 6 weeks, 3, and 6 months was compared between the two groups using Wilcoxon Rank-Sum tests. The change of each marker for each patient at 3 and 6 months from baseline was tested by Wilcoxon Signed-Rank tests. A total of 20 cell types were tested at each time point; however, given the exploratory nature of the analysis, no adjustment for multiple testing was made.

## Results

Long term monitoring in patients with available specimens at 3 (n = 28), 6 (n = 22), and 9 (n = 13) months was performed utilizing multicolor flow cytometry according to the identical SOPs and antibody panels used in the earlier analysis. Among the 13 patients with biospecimens available at the 9 months’ time point, only 2 had relapsed.

**Fig. 2:**
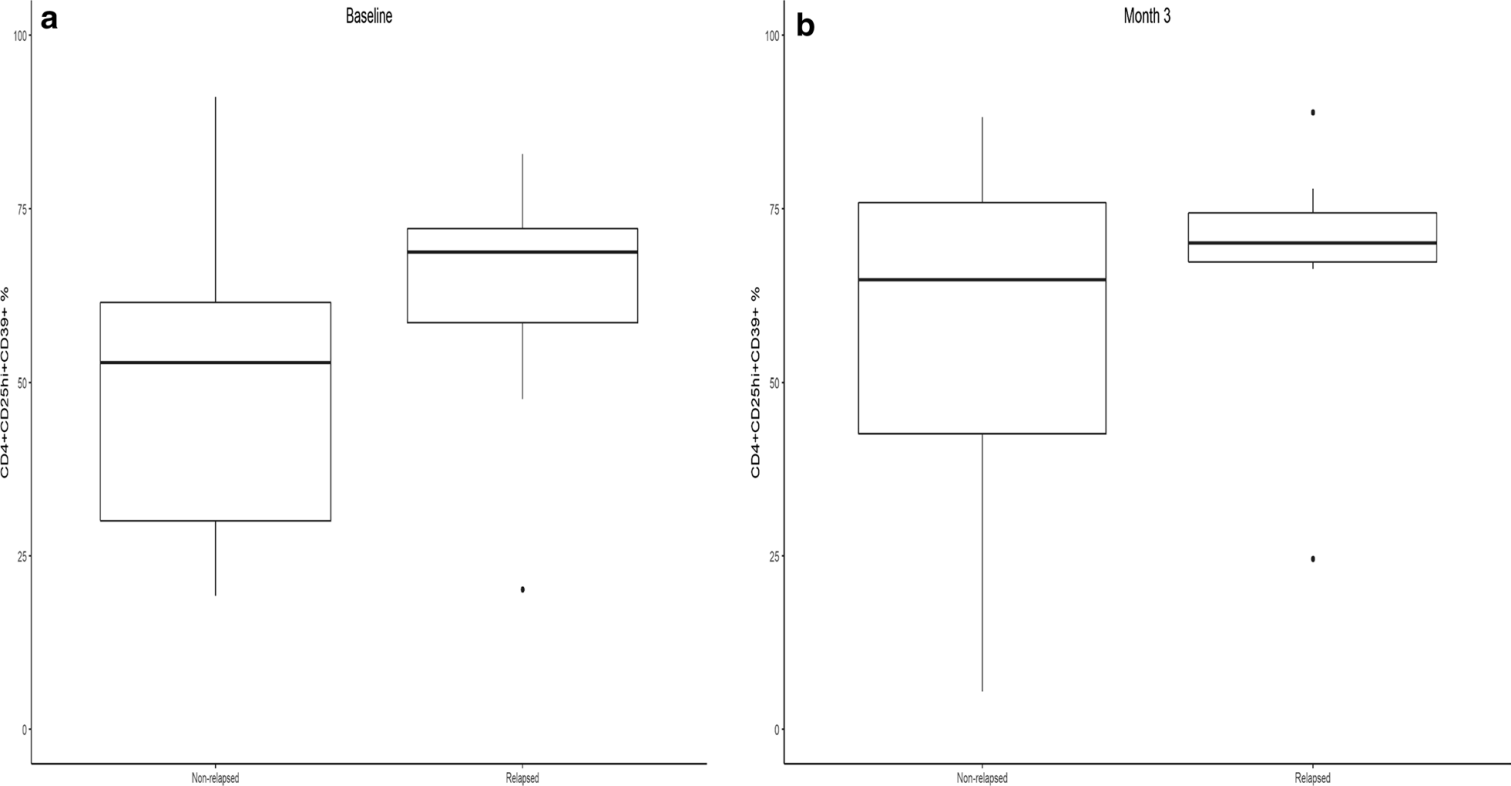
Frequencies of peripheral blood CD4+CD25^hi+^CD39+ Treg at baseline and 3 months in patients who relapsed or were relapse-free at 9 months. **a** There was a significant difference in the percent of CD4+CD25hiCD39+ Treg at baseline between patients who had not relapsed at 9 months versus those who relapsed or died (p = 0.04). Those in the relapse group had significantly higher Treg levels. **b** By month 3, Treg in both groups tended to be higher compared to baseline, but to a greater degree in the relapse-free group. There was no significant difference in Treg levels at month 3 between the two groups (p = 0.266). Y axis shows the % of CD4^+^CD25^hi+^ cells that express CD39

**Fig. 3:**
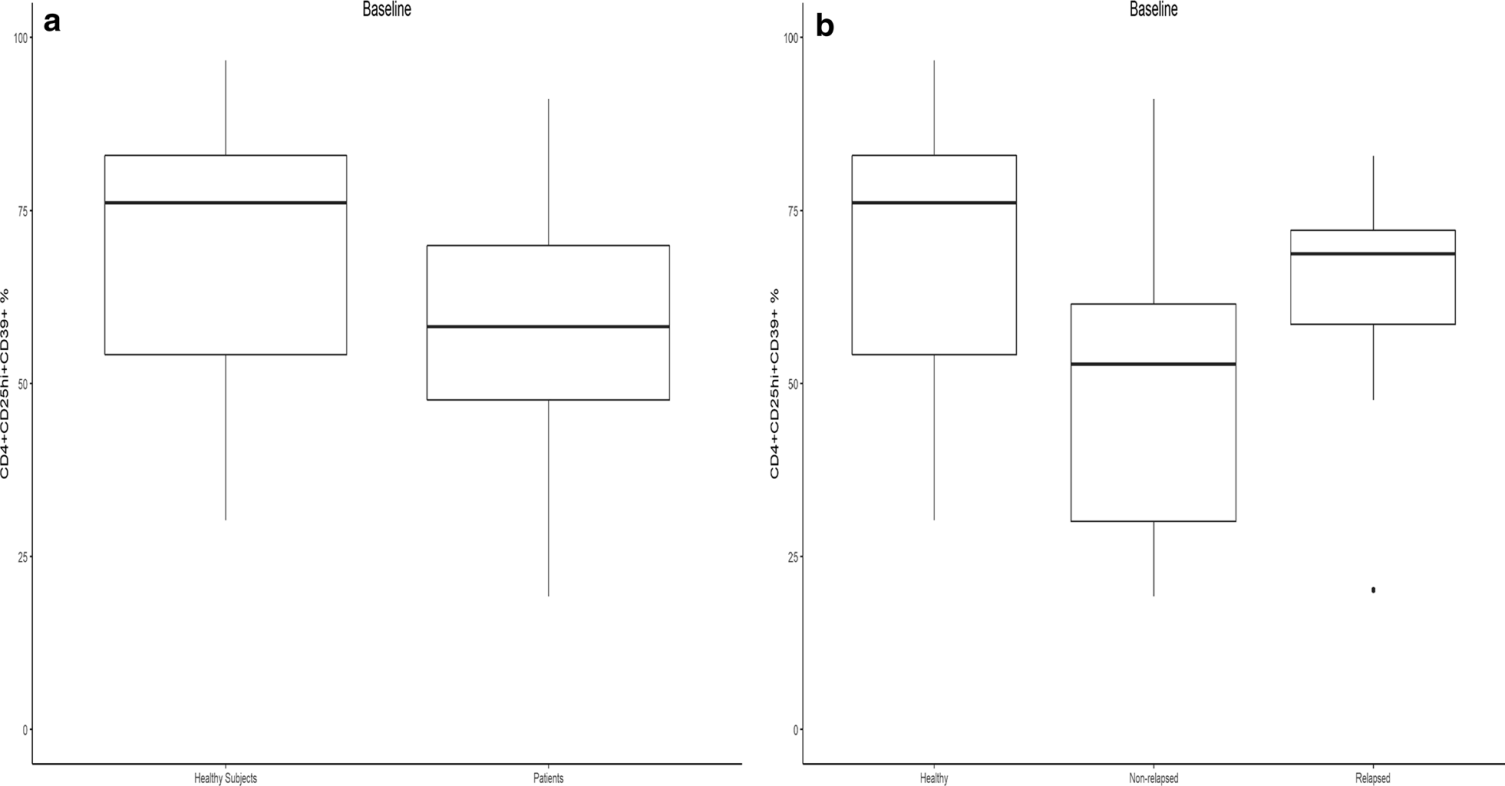
Frequencies of CD4+CD25hiCD39+ Treg at baseline in healthy donors and patients. **a** Median levels of CD39+ Treg at baseline was lower in patients than in healthy donors, but the difference did not reach statistical significance (p = 0.144). **b** Median levels of CD39+ Treg at baseline was highest in healthy donors and lowest in non-relapsed patients, but the difference was not significant

**Fig. 4:**
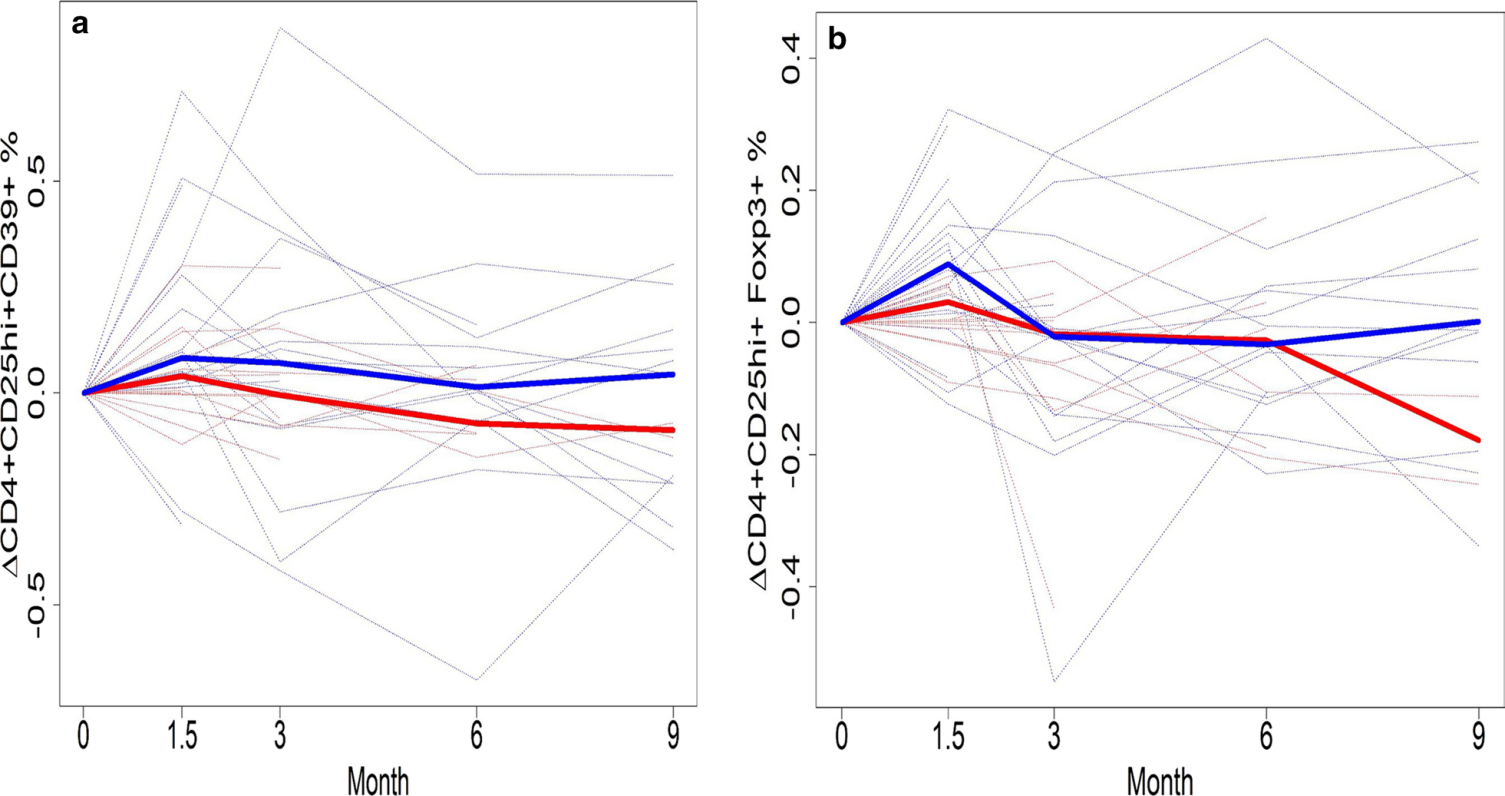
Frequencies of CD4+CD25hiFoxp3+ Treg and CD4+CD25hiCD39+ Treg from baseline through 9 months in patients who did not relapse or relapsed at 9 months. The solid blue line represents the median value of Treg for the relapse-free group, and the solid red line represents the median value for the relapsed group. **a** The levels of CD4+CD25hiFoxp3+ Treg increased at 6 weeks in both groups but returned to almost baseline levels starting 3 months. There were no significant differences in CD4+CD25hiFoxp3+ Treg between the two groups at baseline, 3, 6, or 9 months. **b** At 3 months, levels of CD4+CD25hiCD39+ Treg returned nearly to baseline. There were no significant differences in Treg levels between the two groups starting at 3 months

Levels of Treg significantly increased when measured at 6 weeks following ipilimumab but almost returned to baseline levels by 3 months, with no significant difference in Treg levels between relapsed and relapse-free groups at 3, 6 or 9 months. In our monitoring of Treg, both CD4+CD25hiFoxp3+ and CD4+CD25hiCD39+ Treg phenotypes were examined. While levels of the intracellular transcription factor Foxp3 is an accepted marker for Treg, there is evidence that CD39, which interacts with CD73, may also be critically informative [20]. CD4+CD25hiCD39+ Treg produce adenosine and therefore may possess more immunosuppressive function [21]. Moreover, they are found in increased levels in cancer patients [22]. At baseline, there was a significant difference in CD39+ Treg phenotype between patients remaining relapse-free at 9 months versus those who relapsed or died (p = 0.04). Those in the relapse group had significantly higher Treg levels (Fig. 2a). The median levels of Treg (as well as the lower and upper quartiles) were higher in the relapsed group. By 3 months (Fig. 2b), there was no significant difference in Treg levels between the two groups (p = 0.266). Levels of CD39+ Treg in healthy donors at baseline were compared to those of melanoma patients and then separately to the relapsed and non-relapsed groups. The median levels of CD39+ Treg tended to be lower in patients than in healthy donors, though this difference did not reach statistical significance (Fig. 3a, p = 0.144). Figure 3b shows that median levels of CD39+ Treg was highest in the healthy donors and lowest in the non-relapsed patients. At 3 months, levels of Fox3p+ Treg returned nearly to baseline levels (Fig. 4a). There were no significant differences in Fox3p+ Treg levels between the two groups at later time points. Levels of CD39+ Treg also trended towards baseline levels at 3 months (Fig. 4b). There were likewise no significant differences in levels of CD39+ Treg at 3, 6, or 9 months. Figure 4 illustrates the variation in percent change of Treg over time compared to baseline in both patient groups who relapsed and those who did not.

**Fig. 5:**
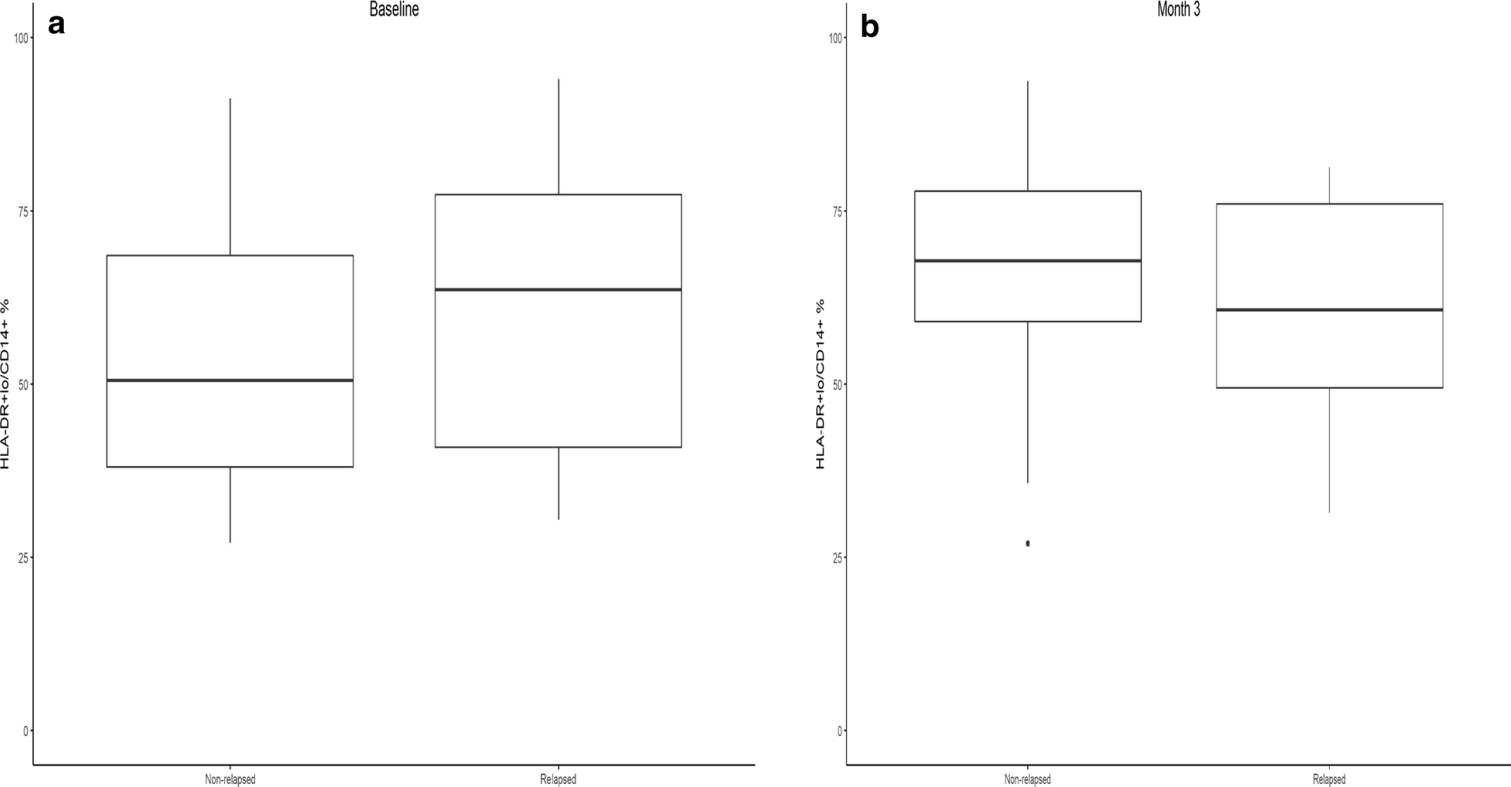
Frequencies of circulating HLA-DR+lowCD14+ MDSC at baseline and 3 months. **a** Levels of circulating monocytic HLA-DR+lowCD14+ MDSC were lower at baseline in the relapse-free group. **b** Median levels increased in the non-relapsed groups at 3 months while staying the same in the relapsed group, but there were no significant differences in levels of MDSC at 3, 6, or 9 months between the 2 groups

Levels of circulating monocytic HLA-DR+/lo CD14+ MDSC were lower at baseline in the relapse-free group but tended to increase at 3 months compared to baseline, though the differences did not reach statistical significance. Levels in the relapsed group remained essentially the same (Fig. 5). There were no significant differences between the 2 groups in levels of MDSC at 3, 6, or 9 months.

**Fig. 6:**
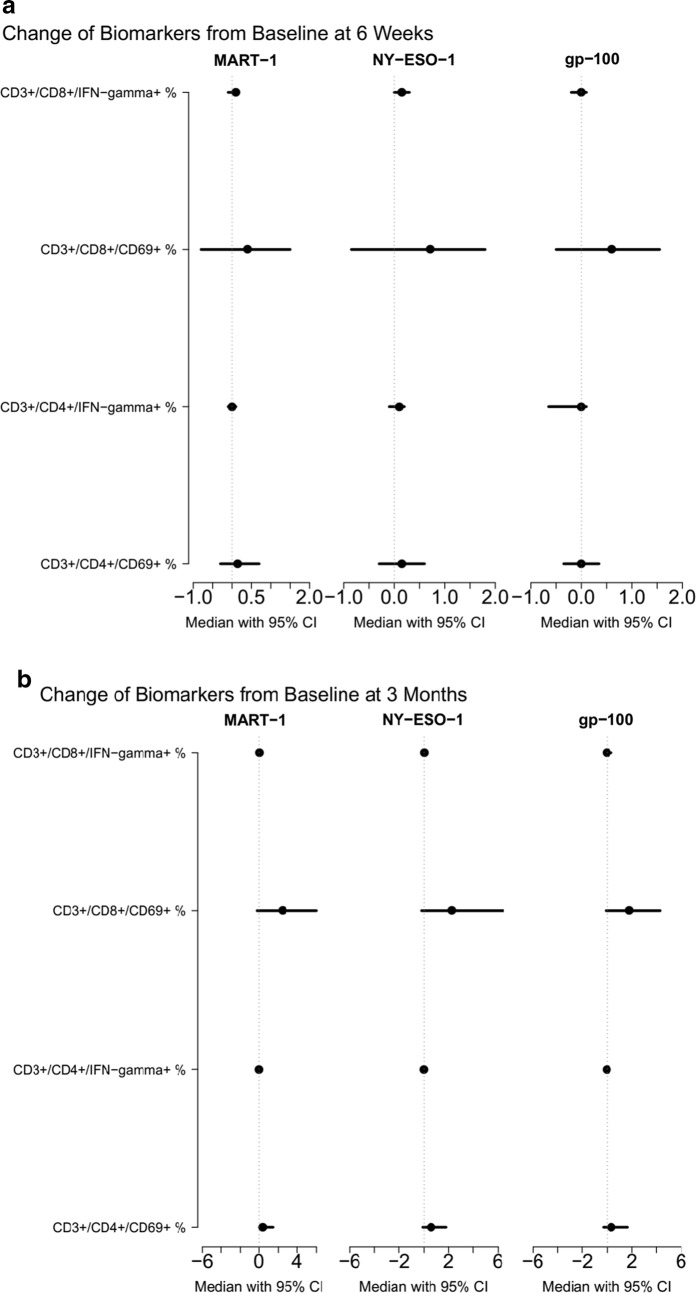
Forest Plots of Type I CD4 and CD8 Ag-specific T Cell Immunity (N = 27) at 6 weeks and at 3 months. Evidence of type I CD4+ and CD8+ antigen-specific T cell immunity against shared melanoma antigens, including lineage antigens (MART-1, gp-100) and cancer testis antigen (NY-ESO1), in the absence of prior vaccination. These responses were boosted after ipilimumab at **a** 6 weeks and **b** 3 months

We detected type I CD4+ and CD8+ antigen-specific T cell immunity against shared melanoma antigens at baseline in the absence of prior vaccination We examined melanocytic lineage antigens (MART-1, gp-100) as well as the cancer testis antigen NY-ESO-1. These responses were potentiated after ipilimumab at 6 weeks and at 3 months (Fig. 6). Both relapsed and relapse-free groups demonstrated significant potentiation of CD4+ and CD8+ T cells specific to shared tumor-associated antigens (gp-100, MART-1, NY-ESO-1). No statistically significant differences in potentiation were seen between relapsed and relapse-free groups.

## Discussion

Tumor immune escape is mediated in part by the recruitment of Treg, which recognize tumor-associated antigens and expand [2]. Treg can also suppress anti-tumor effector and helper T cells in both a tumor-antigen specific and non-specific manner through secretion of soluble factors [23]. Circulating Treg have been shown to be elevated at baseline in PBMCs of advanced melanoma patients compared to healthy donors [24, 25]. Because CTLA-4 is constitutively expressed on CD4+CD25+ Treg, CTLA-4 blockade might be expected to suppress Treg function and allow expansion of a tumor-specific immune response [3]. We previously reported a significant increase in circulating CD4+CD25hi+Foxp3+ and CD4+CD25hi+CD39+ Treg from baseline to 6 weeks following ipilimumab. We unexpectedly found that a greater increase in CD4+CD25hi+Foxp3+ Treg was associated with improved PFS (p = 0.034; HR = 0.57). There was also an association with greater increase in CD4+CD25hi+ T cells (p = 0.043; HR = 0.62) [19]. It was not clear whether the increase in Treg at 6 weeks was a transient or a long term impact of ipilimumab. In the present study with longer patient outcome follow up, we observed significantly higher levels of CD4+CD25hi+CD39+ Treg at baseline in patients who relapsed or died (p = 0.04). By week 6, Treg in both patient groups had increased, but to a greater degree in the relapse-free group. This may explain the previous observation that a greater increase in circulating Treg was associated with improved RFS. At 3 months, overall levels of CD4+CD25hi+FOXP3+ and CD4+CD25hiCD39+ Treg returned nearly to baseline. There were no significant differences in Treg levels between the two groups from 3 months onward. There were likewise no significant differences in levels of CD4+CD25hiFox3p+ Treg at 3, 6, or 9 months.

A number of previous studies reported no change in Treg following ipi, though several observed an increase in activated T responder cells [26–28]. One small study (n = 4) saw a trend toward a relative increase in Treg in some patients [29], whereas another study (n = 10) reported a small significant increase in Treg following tremelimumab [30]. In accordance with our findings, one study found an increase in Treg at 3 weeks followed by a decrease below baseline at week 9 (n = 5) following ipi at 3 or 10 mg/kg [31]. One study reported that change in Treg following 10 mg/kg ipi did not correlate with response [32], while another saw a decrease in Treg associated with increased disease control and survival following treme [33]. We observed no significant difference in baseline PBMC CD39+ Treg levels in healthy subjects compared to all patients or compared to non-relapsed and relapsed patients (Table 2). However, the finding that Treg are significantly higher at baseline in those with clinically poor outcomes despite treatment with ipi suggests that baseline Treg levels, compared to those of other patients with metastatic melanoma, might be prognostic. The additional finding that Treg levels rose in the relapse-free group to a much greater degree than in the relapse group, coupled with the previous finding that a greater increase in circulating Treg was associated with improved PFS, suggests that relative Treg level following ipilimumab may be a predictive indicator early on treatment.

**Table 2:** Healthy donors PBMC T-regulatory cell analysis

Subject	CD4+/CD52hi+Foxp3+%	CD4+/CD25hi+/CD39+%
1	83.7	39.3
2	88.2	30.2
3	80.2	76.9
4	88.2	89.0
5	97.7	76.1
6	89.7	69.0
7	95.9	96.7
Average	89.08	68.17

MDSC are a heterogeneous population of immature myeloid cells associated with tumor immune escape and suppression of the immune response [34–36]. In the tumor microenvironment (TME), these cells are prevented from maturing into dendritic cells or macrophages acting against the tumor; instead, they become tumor-associated macrophages, promoting tumor growth and immune suppression [37]. They have been found in patients with many different cancers and have been associated with more extensive tumor burden and with poor prognosis [38–40]. HLA DR+/lo CD14+ MDSC have been detected specifically in melanoma patients. Studies have shown increased levels, often correlated with tumor burden and prognosis, and evidence of immune suppression [41–47]. Walter et al. tested six predefined populations of MDSC in patients with renal cell cancer treated with the IMA901 vaccine (consisting of multiple tumor-associated peptides) and found 2 to be prognostic for overall survival [4]. MDSC have also been studied in patients receiving CTLA-blockade [48, 49]. An association with decreased MDSC and clinical response was seen in one study [50].

As previously reported, at 6 weeks following ipilimumab, we observed a decrease in percentage of all populations of MDSC from baseline. The greatest decrease was seen for HLA DR+/lo CD14+ MDSC, while a greater decrease in Lin1−/HLA DR−/CD33+/CD11b+ MDSC was associated with PFS (p = 0.03) [19]. As a follow up analysis, it was important investigate what happened to MDSC beyond the initial early-on treatment significant reduction. In the present study, with longer follow up we found that levels of circulating monocytic HLA DR+/lo CD14+ MDSC were lower at baseline in the relapse-free group and further decreased at 6 weeks compared to baseline, though the differences did not reach statistical significance. There were no significant differences in levels of MDSC at 3, 6 months, or 9 months between the relapsed and nonrelapsed groups. A low frequency of CD14+CD11b+HLA-DR−/low MDSC has been previously associated with survival in patients with advanced melanoma [47]. In accord with our previous results, one study found that melanoma patients had increased levels of Lin−CD14+HLA-DR− monocytic MDSC compared to healthy controls, with levels increased with extensive metastases. Patients who responded to ipi clinically had significantly fewer of these MDSC than those who did not respond. Levels did not change significantly from baseline during treatment and did not change following tumor resection [50]. Another study saw a significant decrease in Lin−HLA-DR−/loCD15+CD33+CD11b+ granulocytic MDSC following the first dose of ipi, with low levels persisting at 9 weeks. Levels of CD3−CD19−HLA-DR−/loCD14+ monocytic MDSC remained constant. However, the authors did observe a significant increase in CD14+PD-L1hi monocytic MDSC at 3 weeks, with a return to baseline at 9 weeks. They speculated that early on treatment, activation of T cells triggered by ipi causes an inflammatory response [31]. Circulating monocytic HLA DR+/lo CD14+ MDSC may serve as a potential predictive marker for response to ipi.

Tumor associated antigens have long been a focus of research seeking to develop vaccines that would prompt a vigorous immune response to the tumor. We previously hypothesized that T cell responses to melanoma tumor antigens might be detected following ipi, even in the absence of therapeutic vaccination, and we reported evidence of type I (interferon-gamma producing), fully activated (CD69+) CD4+ and CD8+ antigen-specific T-cell immunity against NY-ESO-1 (cancer-testis), MART-1 and gp100 melanocytic lineage antigens following treatment with ipi at 6 weeks. The largest increases (greater than threefold) in CD3+/CD4+/IFN-gamma+ T cells were observed only in those patients who had not progressed at 6 months [19]. We were interested in evaluating whether there was evidence of a more durable effector T-cell response beyond the initial early-on treatment changes that may support the durable clinical impact of ipilimumab. In the present study, we again observed evidence of spontaneous T cell immunity against shared melanoma antigens potentiated after ipi at 6 weeks and 3 months. Both relapsed and relapse-free groups demonstrated significant potentiation of CD4+ and CD8+ T cells specific to shared tumor-associated antigens (gp-100, MART-1, NY-ESO-1). There were no statistically significant differences in boosting pre-existing responses between relapsed and relapse-free groups starting at 3 months. Several prior studies have also detected an increase in T cell response to the tumor antigen NY-ESO-1 in serum of patients treated with ipi [51–53], sometimes with correlation to clinical benefit [47, 54, 55]. These findings and our own suggest that antigen-specific T cell immunity against shared tumor-associated antigens may be a predictive marker, at least early on treatment. Finally, the potential early-on treatment predictive value of the cellular changes observed warrant further investigation in relation to ipilimumab clinical benefit and these can be validated in larger studies with available biospecimens, individually and in combination with other markers as part of a biomarker signature. The differential impact on circulating Treg, MDSC and effector T cell levels induced by other checkpoint modulators (e.g., anti-PD1) and combinations (e.g., anti-PD1 + anti-CTLA4) relative to CTLA4 blockade alone would be interest in further illuminating the mechanistic impact of these agents and combinations. Since this was a neoadjuvant study with complete tumor resection planned shortly after the initiation of ipilimumab, a limitation of our current report is the inability to investigate the observed longer term cellular changes within the TME including the previously reported induction/potentiation of memory T cells (CD3+/CD8+/CD45RO+TNFα+) in the TME at 6 weeks. Such an evaluation would be of interest in future studies of stage IV patients with serial biopsies, possibly in the second line setting where ipilimumab is currently in clinical use.

## Conclusions

Our long-term monitoring of Treg, MDSC, and tumor antigen response at 3, 6, and 9 months following treatment with ipilimumab resulted in several important findings. First, the significant increase in Treg (CD4+CD25hi+Foxp3+ and CD4+CD25hi+CD39+) at 6 weeks reversed starting 3 months. Second, CD4+CD25hiCD39+ Treg and HLA-DR+lowCD14+ MDSC may be baseline markers of immunotherapeutic benefit and warrant further study. Finally, antigen-specific T cell immunity against shared tumor-associated antigens (gp-100, MART-1, NY-ESO-1) are boosted with CTLA4 blockade and tend to be durable.

## Abbreviations

Treg: regulatory T cell
MDSC: myeloid derived suppressor cells
RFS: relapsed free-survival
IFN: interferon-alfa
Ipi: ipilimumab
OS: overall survival
TME: tumor microenvironment
